# The effect of disease on human cardiac protein expression profiles in paired samples from right and left ventricles

**DOI:** 10.1186/1559-0275-11-34

**Published:** 2014-09-01

**Authors:** Ben Littlejohns, Kate Heesom, Gianni D Angelini, M-Saadeh Suleiman

**Affiliations:** 1 Bristol Heart Institute, School of Clinical Sciences, Faculty of Medicine & Dentistry, University of Bristol, Bristol, UK; 2 Proteomics Facility, Faculty of Medical and Veterinary Sciences, University of Bristol, Bristol, UK

**Keywords:** Proteomics, Human, Cardiac, Coronary artery disease, Aortic valve stenosis, Ventricular biopsies, TMT tag, Mass spectrometry

## Abstract

**Background:**

Cardiac diseases (e.g. coronary and valve) are associated with ventricular cellular remodeling. However, ventricular biopsies from left and right ventricles from patients with different pathologies are rare and thus little is known about disease-induced cellular remodeling in both sides of the heart and between different diseases. We hypothesized that the protein expression profiles between right and left ventricles of patients with aortic valve stenosis (AVS) and patients with coronary artery disease (CAD) are different and that the protein profile is different between the two diseases. Left and right ventricular biopsies were collected from patients with either CAD or AVS. The biopsies were processed for proteomic analysis using isobaric tandem mass tagging and analyzed by reverse phase nano-LC-MS/MS. Western blot for selected proteins showed strong correlation with proteomic analysis.

**Results:**

Proteomic analysis between ventricles of the same disease (intra-disease) and between ventricles of different diseases (inter-disease) identified more than 500 proteins detected in *all relevant* ventricular biopsies. Comparison between ventricles and disease state was focused on proteins with relatively high fold (±1.2 fold difference) and significant (P < 0.05) differences. Intra-disease protein expression differences between left and right ventricles were largely structural for AVS patients and largely signaling/metabolism for CAD. Proteins commonly associated with hypertrophy were also different in the AVS group but with lower fold difference. Inter-disease differences between left ventricles of AVS and CAD were detected in 9 proteins. However, inter-disease differences between the right ventricles of CAD and AVS patients were associated with differences in 73 proteins. The majority of proteins which had a significant difference in one ventricle compared to the other pathology also had a similar trend in the adjacent ventricle.

**Conclusions:**

This work demonstrates for the first time that left and right ventricles have a different proteome and that the difference is dependent on the type of disease. Inter-disease differential expression was more prominent for right ventricles. The finding that a protein change in one ventricle was often associated with a similar trend in the adjacent ventricle for a large number of proteins suggests cross-talk proteome remodeling between adjacent ventricles.

## Background

Studies investigating cardiac gene and protein expression profiling in disease state provide insight into pathophysiological mechanisms and improve our understanding of cardiac cellular remodeling which can help in the development of new therapeutic drugs. Although gene and protein expression profiling have been carried out in hearts of experimental models of disease, these models have significant limitations. For example non-transgenic models of coronary artery disease involve acute ischemia rather than progressive atherosclerotic coronary disease which would trigger cardiac remodeling [1]. In contrast there are several models of ventricular hypertrophy that have been extensively used and do provide useful information. Ideally, gene and protein profiling studies need to be carried out using human tissue. However, studies using human cardiac tissues are rare and have mostly focused on global gene expression in failing and non-failing donor hearts and comparing atrial with ventricular tissue [2-5]. More recently, a comparative study of global gene expression analysis performed on human paired samples collected from the right atrial appendage and from the left ventricle of patients with mixed pathologies (coronary artery bypass graft and aortic valve replacement) identified 542 genes as differentially expressed which corresponded to ∼ 2% of the genes covered by the microarray [6]. Gene expression profiling may not be sufficient to implicate disease progression as protein levels would be more informative. For example a study looking at familial hypertrophic cardiomyopathy caused by a mutation of the beta-myosin heavy chain could not detect differences in the expression of myosin mRNA between left and right ventricles [7]. To the best of our knowledge, there are no studies looking at protein profiling in left and right ventricles of patients with aortic valve stenosis (AVS) and coronary artery disease (CAD).

In this novel study we hypothesized that cardiac diseases trigger different proteomic remodeling in left and right ventricles and that the extent of remodeling varies with disease type. To address this hypothesis we used proteomic analysis on tissues from the right and left ventricles of patients with either AVS or CAD.

## Results

### Comparison between proteomic analysis and western blotting for catalase

**Figure 1:**
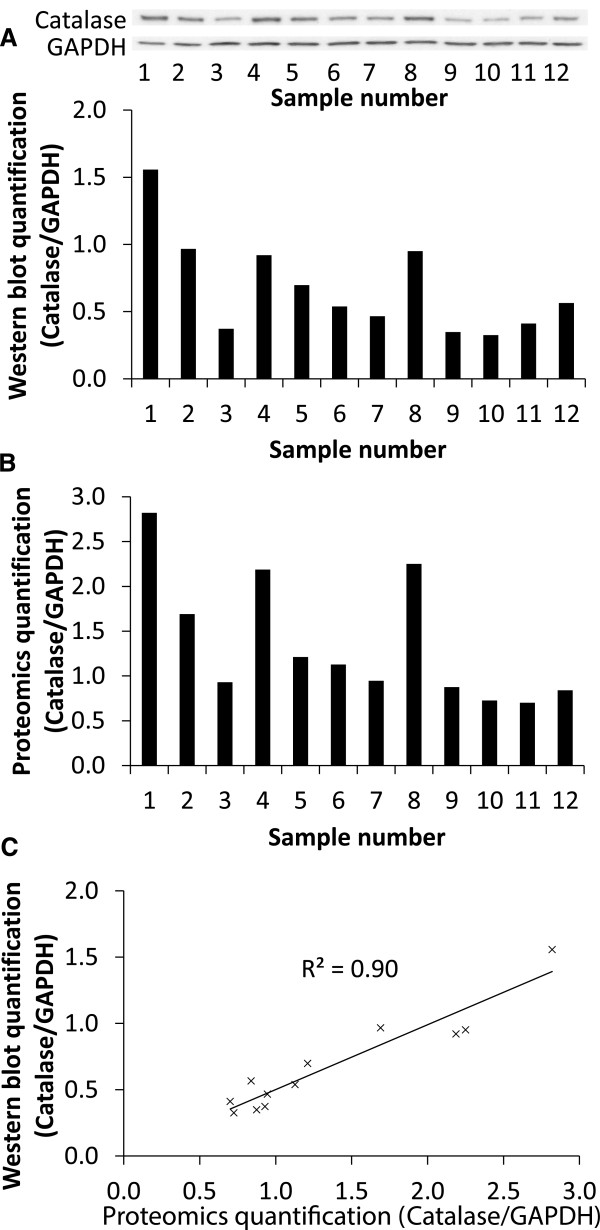
**Comparing proteomics and western blot analysis for catalase. A)** Western blot membranes and quantification of catalase protein expression normalized to GAPDH protein expression. **B)** Proteomics quantification for catalase normalized to GAPDH. The samples used in the western blot were the same samples that were used in the proteomics analysis. **C)** A scatter graph to show the correlation between the quantification of the western blot and the proteomics analysis and a R^2^ value of 0.90 was achieved demonstrating excellent correlation.

To ensure that our proteomic analysis has some validity, we carried out western blotting using the same samples for standard proteins that have available antibodies and are widely used. Catalase and glyceraldehyde-3-phosphate dehydrogenase (GAPDH) expression measured using western blotting showed similar variation to those obtained using proteomic analysis. Samples from both AVS and CAD patients and from both left and right ventricles were selected for western blot analysis. The western blot for catalase and GAPDH and ratios of intensity for the selected samples are shown in Figure 1A. The results compared favorably with the proteomic analysis for catalase and GAPDH as shown in Figure 1B. In fact there was an excellent (R^2^ = 0.90) and significant (P < 0.05) correlation between the western blot analysis and the proteomic analysis for the ratio of catalase/GAPDH (Figure 1C). It must be emphasized however, that this successful validation may not necessarily apply to other proteins or the overall proteomics results.

### The effect of disease on proteomes of paired samples from left and right ventricles of the same patients

#### Aortic valve stenosis

**Figure 2:**
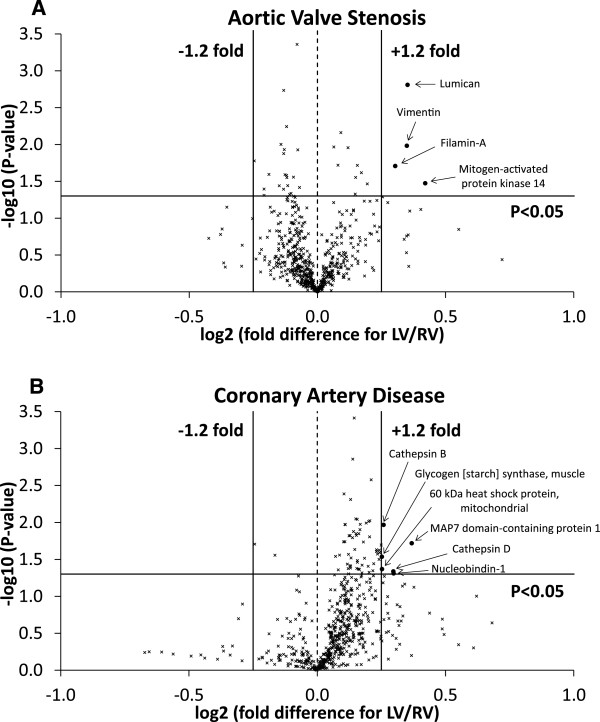
**Protein comparison between the left and right ventricles of each disease. A)** Volcano plot of the entire set of proteins quantified in the left and right ventricles of AVS patients. **B)** Volcano plot of the entire set of proteins quantified in the left and right ventricles of CAD patients. Each point represents the difference in expression (log_2_ fold difference) between left and right ventricles plotted against the level of statistical significance. Solid lines represent ± 1.2 fold difference and a significance level of P < 0.05 (Student’s t-test). Proteins represented by (x) had less than ± 1.2 fold difference or were not statistically significant. Proteins represented by a (•) had greater than ± 1.2 fold difference and were statistically significant.

Overall more than 1700 proteins were detected using this proteomic analysis. However, only 603 proteins were detected in both the right and left ventricles of all AVS patients. In 34 (5.6%) of these proteins there was a significant difference between the two ventricles (Figure 2A). However, there were only four proteins with relatively high fold difference (log_2_ fold difference > 0.25) in the left compared to right ventricle; lumican, vimentin, filamin-A and mitogen-activated protein kinase 14 (Figure 2A). All four proteins were higher in the hypertrophic left ventricle compared to the right ventricle. In addition, proteins commonly associated with hypertrophy were also detected and were significantly lower in the left ventricle compared to the right ventricle but had a relatively low fold difference. These include sarcoplasmic reticulum Ca^2+^ ATPase (log_2_ fold difference = −0.129 and P = 0.01) [8,9] and myosin-binding protein C (log_2_ fold difference = −0.246 and P = 0.02) [10,11].

#### Coronary artery disease

There were 591 proteins detected in both the right and left ventricles of all CAD patients, of which 64 (10.8%) were significantly different between the two ventricles (Figure 2B). However, there were only six proteins (1.0%) with relatively high fold difference (log_2_ fold difference > 0.25) in left compared to right ventricle and they were all higher in the left ventricle (Figure 2B); cathepsins B and D, nucleobindin-1, 60 kDa heat shock protein, glycogen synthase and MAP7 domain-containing protein 1. The volcano plot (Figure 2B) comparing left and right ventricles showed a shift to the right side indicating that there are more proteins with higher expression in left ventricle compared to right ventricle.

### Proteomic analysis comparing ventricles between patients with AVS and patients with CAD

#### Left ventricles

**Figure 3:**
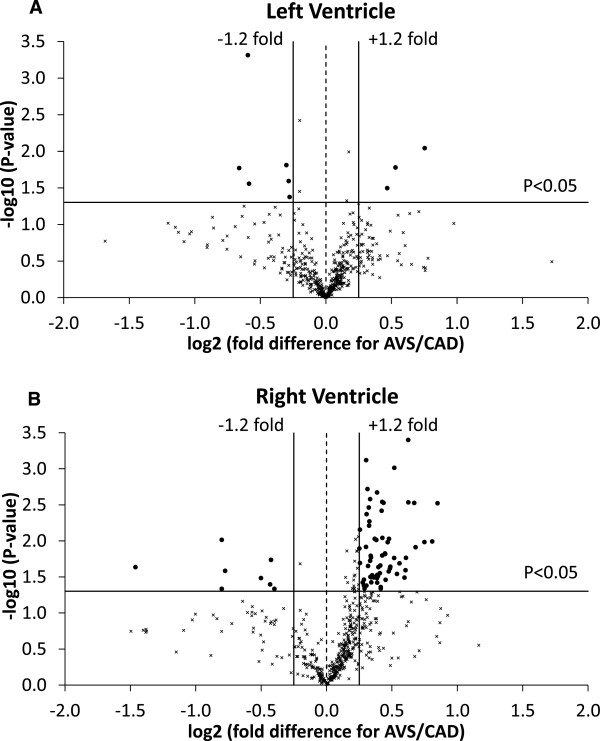
**Protein comparison between CAD and AVS patients. A)** Volcano plot of the entire set of proteins quantified in the left ventricle of CAD and AVS patients. **B)** Volcano plot of the entire set of proteins quantified in the right ventricle of CAD and AVS patients. Each point represents the difference in expression (log_2_ fold difference) between CAD and AVS patients plotted against the level of statistical significance. Solid lines represent ± 1.2 fold difference and a significance level of P < 0.05 (Student’s t-test). Proteins represented by (x) had less than ± 1.2 fold difference or were not statistically significant. Proteins represented by (•) had greater than ± 1.2 fold difference and were statistically significant.

There were 516 proteins detected in all left ventricles of AVS and CAD patients, of which 13 (2.5%) were significantly different between the left ventricle of AVS and the left ventricle of CAD patients (Figure 3A). However, there were only three (0.6%) proteins with relatively high fold difference (log_2_ fold difference > 0.25) which were higher in AVS left ventricle compared to CAD left ventricle, which were: sodium channel protein type 5 subunit alpha, 2-oxoisovalerate dehydrogenase subunit alpha and glycogen synthase (Additional file 1: Table S1). Additionally, there were six (1.2%) proteins which were lower with relatively high fold difference (log_2_ fold difference > 0.25) in the left ventricle of AVS compared to left ventricle of CAD patients: glutathione S-transferase P, myomesin-1 and 2, proactivator polypeptide, apoptotic chromatin condensation inducer in the nucleus and heterogeneous nuclear ribonucleoproteins C1/C2 (Additional file 1: Table S1).

In addition to these proteins that had high fold difference, the proteins that are commonly associated with hypertrophy also showed a strong fold difference but did not reach statistical significance. These include sarcoplasmic reticulum Ca^2+^ ATPase (log_2_ fold difference = −0.243 and P = 0.07) [8,9] and myosin-binding protein C (log_2_ fold difference = −0.691 and P = 0.11) [10,11].

#### Right ventricles

Of the 516 proteins detected in all right ventricles of the AVS and CAD patients there was a significant difference in 92 (17.8%) of proteins (Figure 3B). In the right ventricle of AVS patients there were 73 (14.1%) proteins which were significantly altered and had a log_2_ fold difference > 0.25 compared to right ventricle of CAD patients; of these, 65 were higher and 8 lower in AVS compared to CAD patients. These proteins included 22 that were related to metabolism, 15 that had roles in cell signaling and 11 that were structural proteins (Additional file 1: Table S2-S4).

### Protein expression profiling in left and right ventricles comparing disease related trends

There were four proteins that were significantly altered in *both* the left and right ventricle of the AVS patients compared to *both* the left and right ventricles of CAD patients; glycogen synthase, 2-oxoisovalerate dehydrogenase subunit alpha, sodium channel protein type 5 subunit alpha and apoptotic chromatin condensation inducer in the nucleus (Table 1). There were 26 proteins that were significantly altered in *both* the left and right ventricle of the AVS group compared to *one* of the CAD ventricles (Table 1). Additionally, there were 72 proteins that were significantly altered in *one* of the AVS ventricles compared to *one* of the CAD ventricles and had a similar trend in the adjacent AVS ventricle. The proteins that showed this pattern were divided into groups related to metabolism (Table 2), structural and cell signaling (Table 3) and other (Table 4). The majority (84%) of these 102 proteins that showed a trend to change in both ventricles of one disease compared to the other were higher in the AVS patients.

**Table 1:** **Proteins differentially expressed in *both *** **AVS ventricles compared to at least one CAD ventricle**

	**Log**_**2 **_**fold diff. vs. RV**_**CAD**_		
**Protein [Swiss-Prot accession number]**	**LV**_**CAD**_	**RV**_**AVS**_	**LV**_**AVS**_
**Both ventricles of AVS vs. both ventricles of CAD**			
Glycogen [starch] synthase, muscle [P13807]	0.25*	0.85*^#^	0.72*^#^
2-oxoisovalerate dehydrogenase subunit alpha, mitochondrial [P12694]	0.08	0.54*^#^	0.52*^#^
Sodium channel protein type 5 subunit alpha [Q14524]	−0.10	0.43*^#^	0.65*^#^
Apoptotic chromatin condensation inducer in the nucleus [Q9UKV3]	0.09	−0.50*^#^	−0.51*^#^
**Both ventricles of AVS vs. one ventricle of CAD**			
Unconventional myosin-XVIIIb [Q8IUG5]	0.09	0.81*^#^	0.79*
Nestin [P48681]	0.23	0.60*	0.53*
Thiomorpholine-carboxylate dehydrogenase [Q14894]	0.19	0.52*	0.74*
60 kDa heat shock protein, mitochondrial [P10809]	0.25*	0.48*	0.39*
PDZ and LIM domain protein 5 [Q96HC4]	0.05	0.45*	0.30*
Amine oxidase [flavin-containing] A [P21397]	0.28	0.45*	0.34*
Peptidyl-prolyl cis-trans isomerase F, mitochondrial [P30405]	0.15	0.44*	0.39*
ATP-binding cassette sub-family F member 1 [Q8NE71]	0.06	0.43*^#^	0.27*
Enoyl-CoA delta isomerase 2, mitochondrial [O75521]	0.18	0.39*	0.32*
Hexokinase-1 [P19367]	0.26	0.39*	0.43*
Protein NipSnap homolog 2 [O75323]	0.08	0.33*	0.29*
Phosphoglycerate mutase 1 [P18669]	0.16*	0.33*	0.33*
GTP:AMP phosphotransferase AK4, mitochondrial [P27144]	0.18	0.32*	0.23*
Hydroxyacyl-coenzyme A dehydrogenase, mitochondrial [Q16836]	0.17*	0.31*	0.23*
Aspartate aminotransferase, mitochondrial [P00505]	0.13	0.30*	0.24*
Annexin A11 [P50995]	0.10*	0.30*	0.22*
Glutathione S-transferase kappa 1 [Q9Y2Q3]	0.09	0.28*	0.26*
Elongation factor Tu, mitochondrial [P49411]	0.13*	0.26*	0.22*
Moesin [P26038]	0.03	0.23*^#^	0.21*
Alpha-2-macroglobulin [P01023]	0.37	−0.77*	−0.71*
Alpha-1-acid glycoprotein 1 [P02763]	0.09	−0.80*	−0.69*
Haptoglobin [P00738]	0.28	−1.46*	−1.41*
Elongation factor 1-alpha 2 [Q05639]	−0.07	0.16*^#^	0.09^#^
Peroxiredoxin-1 [Q06830]	−0.04	−0.17^#^	−0.23^#^
Proactivator polypeptide [P07602]	0.24*	−0.33^#^	−0.34^#^
Heterogeneous nuclear ribonucleoproteins C1/C2 [P07910]	0.05	−0.60^#^	−0.61^#^

Proteins significantly altered in both the left and right ventricles of the AVS patients compared to the left and/or right ventricle(s) of CAD patients. Data are presented as log_2_ fold difference compared to the right ventricle of the CAD patients.

*P < 0.05 vs. RV_CAD_ and ^#^P < 0.05 vs. LV_CAD_.

Fold diff. = Fold difference.

**Table 2:** Inter-disease trends: metabolism-related proteins

	**Log**_**2 **_**fold diff. vs. RV**_**CAD**_		
**Protein [Swiss-Prot accession number]**	**LV**_**CAD**_	**RV**_**AVS**_	**LV**_**AVS**_
Succinyl-CoA:3-ketoacid coenzyme A transferase 1, mitochondrial [P55809]	0.14	0.63*^#^	0.37
UTP--glucose-1-phosphate uridylyltransferase [Q16851]	0.03	0.47*^#^	0.37
ADP/ATP translocase 2 [P05141]	0.11	0.42*^#^	0.38
Acyl-coenzyme A thioesterase 9, mitochondrial [Q9Y305]	−0.05	0.39*^#^	0.29
Dihydrolipoyl dehydrogenase, mitochondrial [P09622]	0.12	0.37*^#^	0.28
Cytochrome c [P99999]	0.05	0.31*^#^	0.24
Phosphoglucomutase-1 [P36871]	−0.02	0.15*^#^	0.07
Acetyl-coenzyme A synthetase, cytoplasmic [Q9NR19]	0.07	−0.20*^#^	−0.17
Medium-chain specific acyl-CoA dehydrogenase, mitochondrial [P11310]	0.09	0.42*	0.34
D-beta-hydroxybutyrate dehydrogenase, mitochondrial [Q02338]	0.20	0.41*	0.34
Enoyl-CoA hydratase, mitochondrial [P30084]	0.15*	0.38*	0.26
Dihydrolipoyllysine-residue acetyltransferase component of pyruvate dehydrogenase complex, mitochondrial [P10515]	0.12	0.35*	0.29
Dihydrolipoyllysine-residue succinyltransferase component of 2-oxoglutarate dehydrogenase complex, mitochondrial [P36957]	0.10	0.34*	0.26
Fumarate hydratase, mitochondrial [P07954]	0.12	0.34*	0.25
Amine oxidase [flavin-containing] B [P27338]	0.06	0.25*	0.13
ADP/ATP translocase 1 [P12235]	0.13	0.24*	0.15
Electron transfer flavoprotein subunit beta [P38117]	0.08*	0.22*	0.14
Fatty acid-binding protein, heart [P05413]	0.03	0.21*	0.18
Malate dehydrogenase, cytoplasmic [P40925]	0.05	0.20*	0.13
Pyruvate dehydrogenase protein X component, mitochondrial [O00330]	0.05	0.34^#^	0.27
Glycogen phosphorylase, brain form [P11216]	−0.01	0.29^#^	0.23
Phosphatidate cytidylyltransferase 2 [O95674]	0.01	0.26^#^	0.17
Triosephosphate isomerase [P60174]	0.05	−0.04^#^	−0.03

Proteins (metabolism-related) significantly altered in one of the AVS ventricles compared to either the left or right ventricle of CAD group and had a similar trend in the adjacent AVS ventricle. Data are presented as log_2_ fold difference compared to the right ventricle of the CAD patients.

*P < 0.05 vs. RV_CAD_ and ^#^P < 0.05 vs. LV_CAD_.

Fold diff. = Fold difference.

**Table 3:** Inter-disease trends: structural and cell signaling-related proteins

	**Log**_**2 **_**fold diff. vs. RV**_**CAD**_		
**Protein [Swiss-Prot accession number]**	**LV**_**CAD**_	**RV**_**AVS**_	**LV**_**AVS**_
**Structural**			
Actin, alpha skeletal muscle [P68133]	−0.15	0.75*^#^	0.45
Versican core protein [P13611]	0.06	0.68*^#^	1.04
Tubulin alpha-4A chain [P68366]	0.17	0.56*^#^	0.41
Cofilin-2 [Q9Y281]	0.05	0.52*^#^	0.38
Myopalladin [Q86TC9]	0.02	0.49*^#^	0.42
Talin-2 [Q9Y4G6]	−0.02	−0.42*^#^	−0.37
CLIP-associating protein 1 [Q7Z460]	0.11	0.29*	0.17
Ezrin [P15311]	−0.01	0.25*	0.16
Myosin-9 [P35579]	0.02	0.38	0.34*
LIM domain and actin-binding protein 1 [Q9UHB6]	0.14	0.22	0.46*
Myomesin-2 [P54296]	0.10	−0.05	−0.20^#^
Myomesin-1 [P52179]	0.04	−0.10	−0.24^#^
**Cell Signaling**			
Inactive dual specificity phosphatase 27 [Q5VZP5]	0.02	0.67*^#^	0.61
Protein phosphatase 1 regulatory subunit 7 [Q15435]	0.09	0.42*^#^	0.21
Tight junction protein ZO-1 [Q07157]	0.08	0.40*^#^	0.35
Heat shock protein beta-1 [P04792]	0.06	0.34*^#^	0.26
Heat shock cognate 71 kDa protein [P11142]	0.06	0.22*^#^	0.14
10 kDa heat shock protein, mitochondrial [P61604]	0.24*	0.41*	0.32
Glycogen synthase kinase-3 beta [P49841]	0.05	0.39*	0.32
Apoptosis-inducing factor 1, mitochondrial [O95831]	0.12	0.33*	0.22
Thioredoxin-dependent peroxide reductase, mitochondrial [P30048]	0.12	0.32*	0.22
Calsequestrin-2 [O14958]	0.03	0.31*	0.14
78 kDa glucose-regulated protein [P11021]	0.07	0.29*	0.22
Stress-70 protein, mitochondrial [P38646]	0.13	0.26*	0.21
Heat shock protein beta-7 [Q9UBY9]	−0.09	0.69^#^	0.55
Glutathione S-transferase P [P09211]	0.09	−0.16	−0.19^#^

Proteins (structural and cell signaling-related) significantly altered in one of the AVS ventricles compared to either the left or right ventricle of CAD group and had a similar trend in the adjacent AVS ventricle. Data are presented as log_2_ fold difference compared to the right ventricle of the CAD patients.

*P < 0.05 vs. RV_CAD_ and ^#^P < 0.05 vs. LV_CAD_.

Fold diff. = Fold difference.

**Table 4:** Inter-disease trends: other proteins

	**Log**_**2 **_**fold diff. vs. RV**_**CAD**_		
**Protein [Swiss-Prot accession number]**	**LV**_**CAD**_	**RV**_**AVS**_	**LV**_**AVS**_
Adenylyl cyclase-associated protein 2 [P40123]	0.01	0.49*^#^	0.50
Sarcolemmal membrane-associated protein [Q14BN4]	0.11	0.48*^#^	0.37
Dynamin-like 120 kDa protein, mitochondrial [O60313]	0.15	0.43*^#^	0.31
Calnexin [P27824]	0.10	0.39*^#^	0.20
Popeye domain-containing protein 2 [Q9HBU9]	0.03	0.38*^#^	0.22
CDGSH iron-sulfur domain-containing protein 1 [Q9NZ45]	0.11	0.36*^#^	0.28
Ubiquitin-conjugating enzyme E2 L3 [P68036]	−0.04	0.16*^#^	0.28
Protein Smaug homolog 1 [Q9UPU9]	0.18	0.63*	0.51
Myelin basic protein [P02686]	0.29	0.61*	0.61
Ras-related protein R-Ras2 [P62070]	0.25	0.61*	0.66
BRISC and BRCA1-A complex member 1 [Q9NWV8]	0.23*	0.45*	0.55
28S ribosomal protein S36, mitochondrial [P82909]	0.17	0.34*	0.31
Protein QIL1 [Q5XKP0]	0.14	0.34*	0.26
LIM domain-binding protein 3 [O75112]	0.19	0.33*	0.23
ES1 protein homolog, mitochondrial [P30042]	0.16*	0.29*	0.27
Transitional endoplasmic reticulum ATPase [P55072]	0.06	0.22*	0.12
Anion exchange protein 3 [P48751]	−0.09	−0.40*	−0.26
Complement C3 [P01024]	0.08	−0.80*	−0.67
Cysteine and glycine-rich protein 3 [P50461]	0.01	0.57^#^	0.37
Eukaryotic translation initiation factor 5B [O60841]	−0.10	0.30^#^	0.26
Calpastatin [P20810]	0.34	0.76	0.69*
Protein PBDC1 [Q9BVG4]	0.13	0.26	0.34*
Proline-rich basic protein 1 [E7EW31]	−0.02	−0.26	−0.41*

Other proteins significantly altered in one of the AVS ventricles compared to either the left or right ventricle of CAD group and had a similar trend in the adjacent AVS ventricle. Data are presented as log_2_ fold difference compared to the right ventricle of the CAD patients.

*P < 0.05 vs. RV_CAD_ and ^#^P < 0.05 vs. LV_CAD_.

Fold diff. = Fold difference.

## Discussion

In this novel work we used proteomic analysis involving tandem mass tagging followed by reverse phase nano-liquid chromatography mass spectrometry/mass spectrometry (LC-MS/MS) to compare protein levels between different ventricles from two different diseases: AVS and CAD. Successful strong correlation between proteomic analysis and western blotting for two proteins suggests this proteomic method is valid although more studies are needed before making a firm conclusion.

This work shows for the first time significant intra- and inter-ventricular differences in protein profiling including; i) between left and right ventricles of patients with AVS and CAD, ii) between the left ventricles of the two pathologies, iii) between the right ventricles of the two pathologies and iv) between ventricular tissues of AVS compared to CAD, irrespective of the side of the heart. The pattern of differential abundances for a large number of proteins tended to be similar for the same disease but different between the diseases.

### The main proteins differentially expressed between left and right ventricles of the same heart in patients with AVS are structural

Aortic valve stenosis is associated with left ventricular hypertrophy. Therefore it is expected that there will be significant differences in protein profiling between the hypertrophic left and the relatively normal right ventricles. Surprisingly there were only four proteins that were differentially expressed (significant and greater than 1.2 fold difference) between left and right ventricles. Three of these proteins (lumican, vimentin and filamin-A) were structural proteins and were higher in the hypertrophic left ventricle. Lumican is a small extracellular matrix-localized proteoglycan, produced by cardiac fibroblasts and plays a role in the fibrillogenesis following insults [12,13]. Lumican gene expression was differentially expressed in dilated cardiomyopathy [14] and its precursor was found at higher abundance in the atrial appendage of patients with AVS compared to CAD [15]. Similarly, vimentin is reported to increase in patients with dilated cardiomyopathy which may reflect activation of interstitial cells and fibrosis during the transition to heart failure [16-19]. Elevated filamins play an essential role in the maintenance of cardiac structural integrity and is altered in cardiomyopathies including aortic stenosis [20-22]. It is important to emphasize that these published studies did not compare left and right ventricular tissue. In addition to the three structural proteins, the levels of mitogen-activated protein kinase 14 in the left ventricle was higher which is consistent with its reported role in cardiomyocyte survival pathway in response to pressure overload [23] and in the pathogenesis of dilated cardiomyopathy [24].

In addition, the patients in the AVS group demonstrated changes in proteins commonly associated with hypertrophy (sarcoplasmic reticulum Ca^2+^ ATPase and myosin-binding protein C) confirming the presence of hypertrophy in the left ventricle of these patients; however, it is likely the hypertrophy was mild as the fold difference was relatively low.

### The main proteins differentially expressed between left and right ventricles of the same heart in patients with CAD are signaling related

Despite the presence of chronic coronary atherosclerotic disease, areas distal to main occlusion can still receive sufficient blood flow due to coronary flow reserve and collateralization. However, the severity of the stenosis and the extent of diffuse CAD will determine resting coronary flow or reserve in diseased myocardial segments [25]. Additionally, patients in this study were having bypass grafts for vessels supplying the left and right ventricles in areas distal to the apex where biopsies were collected. Nonetheless, there were several proteins that were significantly more expressed and had relatively high fold difference in the left ventricle compared to right ventricle. These proteins were cathepsins B and D, nucleobindin-1, 60 kDa heat shock protein, glycogen synthase and MAP7 domain-containing protein 1. The difference in the expression of these proteins can be due to differences between left and right ventricles and/or due to severity of disease present.

Cathepsins (cysteine proteases) are implicated in cardiovascular disease and their inhibition is cardioprotective in animal models [26,27] and can degrade unwanted intracellular proteins during ischemic disease [28]. Similarly, the higher level of mitochondrial 60 kDa heat shock protein in left ventricle is cardioprotective against ischemia/reoxygenation [29]. These findings suggest more stress associated with coronary disease in the left compared to right ventricle in view of its higher force generated and therefore more need for energy substrates.

Nucleobindin 1 is a calcium-binding protein and therefore can be involved in calcium storage and signaling [30-33]. This may be required for the relatively higher stress in the left ventricle. The higher MAP7 domain-containing protein 1 in left ventricle compared to right is again likely to be associated with different structure (microtubules) in the thicker left ventricle. Glycogen synthase increased in the left ventricle compared to the right ventricle which implies the left side is storing more glycogen compared to the right or that there is more use of glycogen due to relatively more work by the left ventricle.

### Differential protein expression between the hypertrophic left ventricle of AVS and left ventricle of CAD patients are hypertrophy and disease signaling related proteins

In an earlier study we compared left ventricles of hearts with CAD with left ventricle from hearts with AVS and found that hypertrophic ventricle had significantly higher concentrations of ATP, but lower concentrations of lactate, branched-chain amino acids and alanine [34]. These differences have important implications for energy metabolism and protein turnover in the two pathologies. Comparison between the left ventricles from the two groups of patients have identified nine differentially and significantly expressed proteins. Proteins were either higher (n = 3) or lower (n = 6) in the hypertrophic left ventricle compared to left ventricle of patients with CAD. The three proteins that were higher in AVS patients were; glycogen synthase, sodium channel protein type 5 subunit alpha (Nav1.5) and the mitochondrial 2-oxoisovalerate dehydrogenase subunit alpha. The relatively higher glycogen synthase in hypertrophy is in concert with the expanding myocytes having a greater glycogen content [35]. The relatively higher expression of Nav1.5 has also been seen at the gene expression level in a model of hypertrophy [36]. In contrast there are reports showing that oxidative stress triggers a decrease in the expression of Nav1.5 [37] which is likely to be seen in the left ventricle with atherosclerosis (an inflammatory disease associated with oxidative stress). Therefore it is not clear whether the difference in Nav1.5 expression is due to an increase in hypertrophy, a decrease in CAD patients or a combination of both. The higher expression of mitochondrial 2-oxoisovalerate dehydrogenase subunit alpha may be related to energy production within the mitochondria but 2-oxoisovalerate has been shown to produce little ATP [38].

There were six proteins that had lower expression in hypertrophic compared to CAD left ventricle: myomesin 1 and 2, glutathione S-transferase P, proactivator polypeptide, heterogeneous nuclear ribonucleoproteins C1/C2 and apoptotic chromatin condensation inducer in the nucleus. The myomesins are elastic proteins mostly located in the center of the M-band [39] and may have implications for hypertrophic cardiomyopathy [40]. Glutathione S-transferase P is involved in the regeneration of reduced thiols [41] and therefore the relatively lower levels may indicate disruption to oxidative state in the hypertrophic myocardium. Interestingly the expression of glutathione-S-transferase α3 tends to increase in atherosclerotic coronary arteries compared to control [42]. Proactivator polypeptide also called prosaposin is cleaved into 4 different saposins which stimulate the hydrolysis of sphingolipids, however the heart contains mainly the precursor form [43]. Heterogeneous nuclear ribonucleoproteins are modular proteins characterized by extensive and divergent functions in nucleic acid metabolism. Multiple roles in transcriptional and posttranscriptional regulation enable them to be effective gene expression regulators [44]. Finally, the apoptotic chromatin condensation inducer in the nucleus is a caspase-3-activated protein required for apoptotic chromatin condensation [45] and its lower expression in hypertrophy suggests lower apoptotic activity in the hypertrophic heart or increased apoptosis in the CAD patients.

### 73 proteins in right ventricles of AVS are differentially expressed (89% higher) compared to CAD right ventricle

There was a large number of proteins differentially expressed (significant and greater than 1.2 fold difference) between the right ventricle of AVS and the right ventricle of CAD patients (Additional file 1: Table S2-S4). This is an interesting and surprising finding as the right ventricle is often assumed to be normal in a patient with disease in the left ventricle. The proteins with altered expression included metabolism, cell signaling and structural. Metabolism-related proteins (n = 22) with high-fold difference had higher expression in the AVS right ventricle compared to CAD right ventricle that were mostly mitochondrial. These changes are indicative of higher metabolic activity in the right ventricle of AVS which is surprising as it is widely accepted that only the left ventricle undergoes significant remodeling. What is more surprising is the finding that several structural proteins were also differential higher in the right ventricle of AVS compared to CAD as the AVS right ventricle does not become hypertrophic. Finally, several of the signaling proteins that were differentially expressed in AVS right ventricle are associated with survival and death indicating a relatively more stressed ventricle compared to CAD right ventricle.

### Comparison between two diseases: proteins differentially expressed between ventricles of the two diseases often show a similar trend in the adjacent ventricles

Many of the proteins which demonstrated a difference in one ventricle of one pathology also showed a similar trend in the adjacent ventricle of the same pathology. There were four proteins that had significantly either higher (n = 3) or lower (n = 1) expression in both left and right ventricles of patients with AVS compared to both ventricles of patients with CAD (Table 1). These have already been discussed when comparing left ventricles from both pathologies; glycogen synthase, mitochondrial 2-oxoisovalerate dehydrogenase subunit alpha, sodium channel protein type 5 subunit alpha and apoptotic chromatin condensation inducer in the nucleus.

Additionally there were 26 proteins that showed higher expression in both ventricles of AVS patients compared to one of the ventricles in CAD patients (Table 1). Of particular interest are: hexokinase 1, amine oxidase [flavin-containing] A (also known as monoamine oxidase type A (MAO-A)) and the anti-oxidant enzyme peroxiredoxin-1. Hypertrophic hearts have an increased level of glycolysis [46] which would explain the higher levels of hexokinase. Up regulation of MAO-A in the heart causes oxidative mitochondrial damage and chronic ventricular dysfunction which might then promote the upregulation of the anti-oxidant, peroxiredoxin-1 [47]. In total, there were 102 proteins which demonstrated this trend to change (84% higher in AVS) in both ventricles of one disease compared to the other disease.

### Protein expression and post-translational modifications

Post-translational modifications (PTMs) of proteins are the key step responsible for regulation of protein activity and function [48,49]. Therefore although our proteomes analysis provide an insight into differences between protein expression due to disease and anatomical location, knowledge of PTMs is essential for making firm conclusions as to whether these differences are relevant functionally. PTMs of proteins include phosphorylation, glycosylation, methylation, and acetylation. Recent technological advances are providing the tools for characterisation of PTMs including mass spectrometry to determine the extent of protein phosphorylation (e.g. [50]). Therefore future studies of PTMs are essential for better understanding of the importance of proteome changes in cardiac disease.

## Conclusions

This work demonstrates for the first time that left and right ventricles of patients with either AVS or CAD have a different proteome and that the difference is dependent on the type of disease. Inter-disease differential expression was more prominent for right ventricles compared to the left ventricles. The finding that a protein change in one ventricle was often associated with a similar trend in the adjacent ventricle for a large number of proteins suggests cross-talk proteome remodeling between adjacent ventricles.

## Methods

### Patients

Non-diabetic adult male patients (n = 8) with no prior cardiac surgery were included in the study. Patients were undergoing surgery for either aortic valve replacement (n = 4) with no significant co-existing coronary artery disease or coronary artery bypass grafting (n = 4). None of the patients had current congestive heart failure and they all had good left ventricular function. The New York Heart Association (NYHA) classifications for AVS patients were I (n = 1) and II (n = 3). The NYHA classifications for CAD patients were I (n = 2) and II (n = 2). One AVS patient had preoperative atrial fibrillation. The investigation conforms to the principles outlined in the Declaration of Helsinki. Local hospital ethical approval (ISRCTN84968882) as well as patient consent was obtained.

### Collection of ventricular biopsy

Immediately following the institution of cardiopulmonary bypass, myocardial tissue biopsy specimens were collected from the apex of the right and the left ventricles using a 14 Ga. TW’11.4 cm cannula Trucut needle (Baxter Healthcare Corporation, USA). Each specimen was immediately snap frozen (less than 5 s) in liquid nitrogen and stored at −80°C until processing for protein extraction and quantification.

### Protein extraction and quantification

Proteins were extracted from ventricular samples using mirVana^TM^ PARIS^TM^ RNA and protein purification kit (Life Technologies, UK). Following extraction, samples were incubated on ice for 30 min and then centrifuged at 10,000 × g for 10 min. The supernatant was collected and stored at −80°C. Protein content was determined using the Bradford assay and samples were diluted to 2 mg · ml^−1^. A pooled sample was prepared by mixing an equal volume of each sample and was used as an internal standard for normalization of each proteomics analysis.

### Proteomics

Analysis of proteins in ventricular tissues was performed by the University of Bristol Proteomics Facility using tandem mass tags (TMTs) (Thermo Fisher Scientific, UK).

#### Tandem mass tag labeling

Aliquots of each sample (100 μg) were reduced with 10 mM TCEP (incubated at 55°C for 1), alkylated with 17 mM iodoacetamide (incubated at room temperature for 30 min) and then digested with trypsin (2.5 μg trypsin per 100 μg protein; 37°C, overnight) and the resulting peptides labeled with TMT sixplex reagents, all according to the manufacturer’s protocol (Thermo Fisher Scientific, UK). Each sample was labeled with a different isobaric tag (6 per experimental comparison) and the tagged samples were then combined.

#### Nano-liquid chromatography mass spectrometry/mass spectrometry

The combined sample was fractionated using a Dionex Ultimate 3000 nano high-performance liquid chromatography system in line with an LTQ-Orbitrap Velos mass spectrometer (Thermo Scientific, UK). In brief, peptides in 1% (v/v) formic acid were injected onto an Acclaim PepMap C18 nano-trap column (Dionex, USA). After washing with 0.5% (v/v) acetonitrile 0.1% (v/v) formic acid peptides were resolved on a 250 mm × 75 μm Acclaim PepMap C18 reverse phase analytical column (Dionex, USA) over a 150 min organic gradient, using 7 gradient segments (1-6% solvent B over 1 min, 6-15% B over 58 min, 15-32% B over 58 min, 32-40% B over 3 min, 40-90% B over 1 min, held at 90% B for 6 min and then reduced to 1% B over 1 min) with a flow rate of 300 nL · min^−1^. Solvent A was 0.1% formic acid and solvent B was aqueous 80% acetonitrile in 0.1% formic acid. Peptides were ionized by nano-electrospray ionization at 2.0 kV using a stainless steel emitter with an internal diameter of 30 μm (Thermo Scientific, UK) and a capillary temperature of 250°C. Tandem mass spectra were acquired using an LTQ-Orbitrap Velos mass spectrometer controlled by Xcalibur 2.1 software (Thermo Scientific, UK) and operated in data-dependent acquisition mode. The Orbitrap was set to analyze the survey scans at 60,000 resolution (at m/z 400) in the mass range m/z 300 to 1800 and the top ten multiply charged ions in each duty cycle selected for MS/MS fragmentation using higher-energy collisional dissociation with normalized collision energy of 45%, activation time of 0.1 ms and at a resolution of 7500 within the Orbitrap. Charge state filtering, where unassigned precursor ions were not selected for fragmentation, and dynamic exclusion (repeat count 1; repeat duration 30 s; exclusion list size 500) were used.

The raw data files were processed and quantified using Proteome Discoverer software v1.2 (Thermo Scientific, UK) and searched against the reviewed Swiss-Prot human database (20,243 entries) using the SEQUEST (Ver. 28 Rev. 13) algorithm. Peptide precursor mass tolerance was set at 10 ppm, and MS/MS tolerance was set at 20mmu. Search criteria included oxidation of methionine (+15.9949) as a variable modification and carbamidomethylation of cysteine (+57.0214) and the addition of the TMT sixplex mass tag (+229.163 Da) to peptide N-termini and lysine as fixed modifications. Searches were performed with full tryptic digestion and a maximum of one missed cleavage was allowed. The reverse database search option was enabled and all peptide data was filtered to satisfy false discovery rate (FDR) of 5%. The Proteome Discoverer software generates a reverse “decoy” database from the same protein database and any peptides passing the initial filtering parameters that were derived from this decoy database are defined as false positive identifications. The minimum cross-correlation factor filter was readjusted for each individual charge state separately to optimally meet the predetermined target FDR of 5% based on the number of random false positive matches from the reverse decoy database. Thus each data set has its own passing parameters. Quantitation was done using a peak integration window tolerance setting of 0.0075 Da with the integration method set as the most confident centroid.

### Western blotting

Extracted proteins (10 μg) were separated using SDS-polyacrylamide gel electrophoresis under reducing and denaturing conditions and transferred to a 0.45 μm polyvinylidene difluoride membrane. The membranes were blocked with tris-buffered saline (TBS)-Tween containing 10% (w/v) skimmed milk powder before incubation overnight (4°C) with a primary antibody diluted in TBS-Tween containing 5% (w/v) BSA. Primary antibodies used included catalase (1:2000, Abcam) and GAPDH (1:10,000, Cell Signaling). Membranes were then incubated with an anti-rabbit horseradish peroxidase conjugated secondary antibody (1:10,000, GE Healthcare Life Sciences) and proteins were visualized using the enhanced chemiluminescence system (Amersham). Protein bands were quantified by densitometry with ImageJ 1.46r software.

### Data analysis

For proteomics analysis protein ratios represent the median of the measured peptide ratio(s) for each. Using this quantitative proteomic approach, the data analysis was set so that any missing reporter ions (TMT tags) in each MS/MS spectra are replaced with a minimum intensity value, such that a protein ratio value is generated even if that protein is not detected in one of the samples in a single run (sixplex) of proteomic analysis. In the current study the maximum fold change was set to 100, meaning that any protein showing a protein ratio of 100 was not detected in one of the samples under comparison. No such proteins were identified. Values for each identified protein were presented as a ratio to the internal standard and then normalized to GAPDH measured using proteomics. Volcano plots were created by plotting log_2_ (fold difference) on the horizontal axis and –log_10_ (P-value) on the vertical axis [51]. A log_2_ fold difference of more than 0.25 (±1.2 fold difference) was considered as high-fold difference and was used as the main focus for presenting and discussing the data. We opted for the relatively low cutoff as the majority of the highly significant changes could be accommodated at around 1.2 fold higher (or lower). Other proteomic studies have also used similar cut-off values [52-54]. Other data are presented as mean ± SEM. All statistical tests were performed as two-tailed and a P-value less than 0.05 was assumed to be significantly different.

## Abbreviations

AVS: Aortic valve stenosis; CAD: Coronary artery disease; GAPDH: Glyceraldehyde-3-phosphate dehydrogenase; LC-MS/MS: Liquid chromatography mass spectrometry/mass spectrometry; MAO-A: Monoamine oxidase type A; PTM: Post-translational modifications; NYHA: New York Heart Association; TMT: Tandem mass tags; FDR: False discovery rate; TBS: Tris-buffered saline; SEM: Standard error of the mean.

## Competing interests

The authors declare that they have no competing interests.

## Authors’ contributions

BL, KH: carried out the work and processed the data. MSS, GDA, BL conceived and designed the work, MSS, BL wrote the manuscript. All authors read and approved the final manuscript.

## Supplementary Material

Additional file 1: Table S1-S4. Proteins differentially expressed between the ventricles of AVS and CAD patients. Click here for file
