# Supplementary material for: The effect of disease on human cardiac protein expression profiles in paired samples from right and left ventricles
Source: Clin Proteomics. 2014 Sep 1;11(1):34. doi: 10.1186/1559-0275-11-34 (PMC4158351; doi:10.1186/1559-0275-11-34)
Supplement: Additional file 1: Table S1-S4. — Proteins differentially expressed between the ventricles of AVS and CAD patients. [file 1559-0275-11-34-S1.docx]

# **Additional files**

## Additional file 1: Table S1 Proteins differentially expressed between the *left ventricles* of AVS and CAD patients.

| **Protein [Swiss-Prot accession number]** | **LV_AVS_** | **LV_CAD_** | **Fold diff. LV_AVS_/LV_CAD_** | **P-Value** |
| --- | --- | --- | --- | --- |
| 2-oxoisovalerate dehydrogenase subunit alpha, mitochondrial [P12694] | 0.921±0.057 | 0.637±0.132 | 1.45 | 0.017 |
| Sodium channel protein type 5 subunit alpha [Q14524] | 1.115±0.164 | 0.661±0.148 | 1.69 | 0.009 |
| Glycogen [starch] synthase, muscle [P13807] | 1.158±0.136 | 0.837±0.109 | 1.38 | 0.032 |
| Glutathione S-transferase P [P09211] | 0.856±0.096 | 1.036±0.032 | 0.83 | 0.042 |
| Myomesin-1 [P52179] | 0.952±0.046 | 1.158±0.098 | 0.82 | 0.026 |
| Myomesin-2 [P54296] | 1.029±0.034 | 1.267±0.116 | 0.81 | 0.016 |
| Proactivator polypeptide [P07602] | 0.812±0.193 | 1.218±0.101 | 0.67 | 0.028 |
| Apoptotic chromatin condensation inducer in the nucleus [Q9UKV3] | 0.823±0.102 | 1.242±0.149 | 0.66 | < 0.001 |
| Heterogeneous nuclear ribonucleoproteins C1/C2 [P07910] | 0.845±0.146 | 1.336±0.191 | 0.63 | 0.017 |

Proteins which had an altered expression level and relatively high fold difference between the left ventricle of AVS patients and the left ventricle of CAD patients. Data are presented as a ratio to the pooled sample, are normalized to GAPDH and are displayed as mean ± SEM. Fold diff. = Fold difference.

## Additional file 1: Table S2 Proteins (metabolism-related) differentially expressed between *right ventricles* of AVS and CAD patients.

| **Protein [Swiss-Prot accession number]** | **RV_AVS_** | **RV_CAD_** | **Fold diff. RV_AVS_/RV_CAD_** | **P-Value** |
| --- | --- | --- | --- | --- |
| Glycogen [starch] synthase, muscle [P13807] | 1.267±0.223 | 0.703±0.125 | 1.80 | 0.003 |
| Succinyl-CoA:3-ketoacid coenzyme A transferase 1, mitochondrial [P55809] | 1.004±0.105 | 0.650±0.121 | 1.54 | 0.003 |
| 2-oxoisovalerate dehydrogenase subunit alpha, mitochondrial [P12694] | 0.933±0.138 | 0.642±0.072 | 1.45 | 0.029 |
| Thiomorpholine-carboxylate dehydrogenase [Q14894] | 0.887±0.070 | 0.620±0.115 | 1.43 | 0.017 |
| UTP--glucose-1-phosphate uridylyltransferase [Q16851] | 1.060±0.102 | 0.766±0.091 | 1.38 | 0.010 |
| Amine oxidase [flavin-containing] A [P21397] | 1.027±0.107 | 0.752±0.068 | 1.37 | 0.015 |
| ADP/ATP translocase 2 [P05141] | 0.940±0.085 | 0.701±0.066 | 1.34 | 0.004 |
| Medium-chain specific acyl-CoA dehydrogenase, mitochondrial [P11310] | 1.064±0.109 | 0.798±0.094 | 1.33 | 0.046 |
| D-beta-hydroxybutyrate dehydrogenase, mitochondrial [Q02338] | 0.929±0.087 | 0.700±0.086 | 1.33 | 0.028 |
| Acyl-coenzyme A thioesterase 9, mitochondrial [Q9Y305] | 1.056±0.038 | 0.804±0.131 | 1.31 | 0.031 |
| Enoyl-CoA delta isomerase 2, mitochondrial [O75521] | 1.014±0.084 | 0.775±0.066 | 1.31 | 0.002 |
| Hexokinase-1 [P19367] | 0.898±0.079 | 0.687±0.085 | 1.31 | 0.033 |
| Enoyl-CoA hydratase, mitochondrial [P30084] | 1.033±0.081 | 0.792±0.086 | 1.31 | 0.031 |
| Dihydrolipoyl dehydrogenase, mitochondrial [P09622] | 1.021±0.083 | 0.791±0.058 | 1.29 | 0.009 |
| Dihydrolipoyllysine-residue acetyltransferase component of pyruvate dehydrogenase complex, mitochondrial [P10515] | 0.968±0.083 | 0.759±0.066 | 1.27 | 0.030 |
| Dihydrolipoyllysine-residue succinyltransferase component of 2-oxoglutarate dehydrogenase complex, mitochondrial [P36957] | 0.990±0.091 | 0.780±0.048 | 1.27 | 0.032 |
| Fumarate hydratase, mitochondrial [P07954] | 1.008±0.096 | 0.797±0.042 | 1.27 | 0.016 |
| Phosphoglycerate mutase 1 [P18669] | 0.975±0.074 | 0.776±0.057 | 1.26 | 0.005 |
| Cytochrome c [P99999] | 1.033±0.068 | 0.831±0.058 | 1.24 | 0.002 |
| Hydroxyacyl-coenzyme A dehydrogenase, mitochondrial [Q16836] | 1.022±0.062 | 0.826±0.055 | 1.24 | 0.004 |
| Aspartate aminotransferase, mitochondrial [P00505] | 1.014±0.082 | 0.822±0.043 | 1.23 | 0.012 |
| Amine oxidase [flavin-containing] B [P27338] | 0.979±0.062 | 0.822±0.045 | 1.19 | 0.013 |

Metabolism-related proteins which had an altered expression level and relatively high fold difference between the right ventricle of AVS patients and the right ventricle of CAD patients. Data are presented as a ratio to the pooled sample, are normalized to GAPDH and displayed as mean ± SEM. Fold diff. = Fold difference.

## Additional file 1: Table S3 Proteins (structural and cell signaling-related) differentially expressed between *right ventricles* of AVS and CAD patients.

| **Protein [Swiss-Prot accession number]** | **RV_AVS_** | **RV_CAD_** | **Fold diff. RV_AVS_/RV_CAD_** | **P-Value** |
| --- | --- | --- | --- | --- |
| **Structural** | | | | |
| Unconventional myosin-XVIIIb [Q8IUG5] | 1.022±0.124 | 0.584±0.158 | 1.75 | 0.010 |
| Actin, alpha skeletal muscle [P68133] | 1.197±0.187 | 0.712±0.144 | 1.68 | 0.010 |
| Versican core protein [P13611] | 0.855±0.116 | 0.533±0.110 | 1.60 | 0.012 |
| Nestin [P48681] | 0.977±0.156 | 0.646±0.088 | 1.51 | 0.032 |
| Tubulin alpha-4A chain [P68366] | 1.023±0.112 | 0.694±0.117 | 1.47 | 0.021 |
| Cofilin-2 [Q9Y281] | 1.013±0.095 | 0.707±0.092 | 1.43 | < 0.001 |
| Myopalladin [Q86TC9] | 0.944±0.086 | 0.673±0.077 | 1.40 | 0.023 |
| PDZ and LIM domain protein 5 [Q96HC4] | 1.108±0.150 | 0.809±0.060 | 1.37 | 0.035 |
| CLIP-associating protein 1 [Q7Z460] | 1.014±0.077 | 0.831±0.063 | 1.22 | 0.035 |
| Talin-2 [Q9Y4G6] | 0.923±0.077 | 1.237±0.118 | 0.75 | 0.018 |
| Lumican [P15884] | 0.796±0.122 | 1.072±0.050 | 0.74 | 0.040 |
| **Cell Signaling** | | | | |
| Inactive dual specificity phosphatase 27 [Q5VZP5] | 1.163±0.135 | 0.730±0.139 | 1.59 | 0.003 |
| 60 kDa heat shock protein, mitochondrial [P10809] | 1.008±0.125 | 0.723±0.056 | 1.39 | 0.009 |
| Protein phosphatase 1 regulatory subunit 7 [Q15435] | 1.064±0.093 | 0.797±0.102 | 1.33 | 0.044 |
| 10 kDa heat shock protein, mitochondrial [P61604] | 1.005±0.105 | 0.755±0.071 | 1.33 | 0.022 |
| Tight junction protein ZO-1 [Q07157] | 1.023±0.105 | 0.777±0.074 | 1.32 | 0.023 |
| Glycogen synthase kinase-3 beta [P49841] | 1.030±0.118 | 0.787±0.059 | 1.31 | 0.038 |
| Heat shock protein beta-1 [P04792] | 0.915±0.083 | 0.723±0.041 | 1.27 | 0.017 |
| Apoptosis-inducing factor 1, mitochondrial [O95831] | 1.025±0.081 | 0.813±0.049 | 1.26 | 0.003 |
| Thioredoxin-dependent peroxide reductase, mitochondrial [P30048] | 1.044±0.075 | 0.833±0.051 | 1.25 | 0.003 |
| Calsequestrin-2 [O14958] | 0.941±0.097 | 0.761±0.029 | 1.24 | 0.042 |
| Annexin A11 [P50995] | 1.018±0.062 | 0.825±0.057 | 1.23 | < 0.001 |
| 78 kDa glucose-regulated protein [P11021] | 0.992±0.070 | 0.813±0.059 | 1.22 | 0.042 |
| Glutathione S-transferase kappa 1 [Q9Y2Q3] | 1.021±0.056 | 0.842±0.066 | 1.21 | 0.038 |
| Stress-70 protein, mitochondrial [P38646] | 1.016±0.069 | 0.851±0.045 | 1.19 | 0.020 |
| Apoptotic chromatin condensation inducer in the nucleus [Q9UKV3] | 0.826±0.069 | 1.168±0.156 | 0.71 | 0.033 |

Structural and cell signaling-related proteins which had an altered expression level and relatively high fold difference between the right ventricle of AVS patients and the right ventricle of CAD patients. Data are presented as a ratio to the pooled sample, are normalized to GAPDH and are displayed as mean ± SEM. Fold diff. = Fold difference.

## Additional file 1: Table S4 Other proteins differentially expressed between the *right ventricles* of AVS and CAD patients.

| **Protein [Swiss-Prot accession number]** | **RV_AVS_** | **RV_CAD_** | **Fold diff. RV_AVS_/RV_CAD_** | **P-Value** |
| --- | --- | --- | --- | --- |
| Protein Smaug homolog 1 [Q9UPU9] | 1.001±0.110 | 0.649±0.105 | 1.54 | < 0.001 |
| Myelin basic protein [P02686] | 1.035±0.095 | 0.679±0.155 | 1.53 | 0.017 |
| Ras-related protein R-Ras2 [P62070] | 0.821±0.101 | 0.540±0.094 | 1.52 | 0.026 |
| Adenylyl cyclase-associated protein 2 [P40123] | 0.965±0.097 | 0.689±0.086 | 1.40 | 0.024 |
| Sarcolemmal membrane-associated protein [Q14BN4] | 0.954±0.081 | 0.685±0.096 | 1.39 | 0.027 |
| BRISC and BRCA1-A complex member 1 [Q9NWV8] | 0.949±0.088 | 0.695±0.093 | 1.36 | 0.015 |
| Peptidyl-prolyl cis-trans isomerase F, mitochondrial [P30405] | 1.026±0.093 | 0.758±0.076 | 1.35 | 0.003 |
| Dynamin-like 120 kDa protein, mitochondrial [O60313] | 1.025±0.108 | 0.762±0.068 | 1.35 | 0.009 |
| ATP-binding cassette sub-family F member 1 [Q8NE71] | 1.045±0.121 | 0.778±0.052 | 1.34 | 0.016 |
| Sodium channel protein type 5 subunit alpha [Q14524] | 0.955±0.065 | 0.711±0.089 | 1.34 | 0.003 |
| Calnexin [P27824] | 0.964±0.089 | 0.736±0.074 | 1.31 | 0.030 |
| Popeye domain-containing protein 2 [Q9HBU9] | 0.983±0.083 | 0.755±0.070 | 1.30 | 0.010 |
| CDGSH iron-sulfur domain-containing protein 1 [Q9NZ45] | 0.983±0.109 | 0.763±0.038 | 1.29 | 0.032 |
| Protein QIL1 [Q5XKP0] | 0.994±0.082 | 0.783±0.067 | 1.27 | 0.038 |
| 28S ribosomal protein S36, mitochondrial [P82909] | 1.007±0.092 | 0.797±0.056 | 1.26 | 0.032 |
| LIM domain-binding protein 3 [O75112] | 0.997±0.069 | 0.791±0.061 | 1.26 | 0.019 |
| Protein NipSnap homolog 2 [O75323] | 0.983±0.051 | 0.784±0.072 | 1.25 | 0.006 |
| GTP:AMP phosphotransferase AK4, mitochondrial [P27144] | 1.029±0.104 | 0.826±0.024 | 1.25 | 0.022 |
| ES1 protein homolog, mitochondrial [P30042] | 0.979±0.057 | 0.800±0.064 | 1.22 | 0.046 |
| Elongation factor Tu, mitochondrial [P49411] | 0.995±0.060 | 0.832±0.048 | 1.19 | 0.007 |
| Anion exchange protein 3 [P48751] | 0.827±0.104 | 1.090±0.106 | 0.76 | 0.046 |
| Alpha-2-macroglobulin [P01023] | 0.821±0.246 | 1.404±0.186 | 0.58 | 0.026 |
| Alpha-1-acid glycoprotein 1 [P02763] | 0.764±0.184 | 1.331±0.192 | 0.57 | 0.010 |
| Complement C3 [P01024] | 0.737±0.276 | 1.284±0.143 | 0.57 | 0.047 |
| Haptoglobin [P00738] | 0.467±0.341 | 1.284±0.244 | 0.36 | 0.023 |

Other proteins which had an altered expression level and relatively high fold difference between the right ventricle of AVS patients and the right ventricle of CAD patients. Data are presented as a ratio to the pooled sample, are normalized to GAPDH and are displayed as mean ± SEM. Fold diff. = Fold difference.
